# Guillain-Barré syndrome in a Child with Ongoing Viral Hepatitis A

**Published:** 2018

**Authors:** Ivan BALTADZHIEV, Ina GENEVA, Nedialka POPIVANOVA

**Affiliations:** 1 Department. of Infectious Diseases, Parasitology, and Tropical Medicine, Medical University and University Clinic of Infectious Diseases, Plovdiv, Bulgaria; 2 Department of Pediatrics, Medical University and University Clinic of Pediatrics, Plovdiv, Bulgaria

**Keywords:** Guillain-Barré syndrome, Viral hepatitis A, Children, Acute inflammatory demyelinating polyneuropathy (AIDP)

## Abstract

Guillain-Barré syndrome (GBS) belongs to the group of peripheral immune-mediated neuropathies often preceded by an inflammatory episode. GBS is rarely associated with hepatitis A virus (HAV) infection, the latter as a rule antecedent of the neurological disorders. This association is quite rare in childhood, and so far, only isolated cases have been described. We report an unusual case of pediatric GBS which development coincided with the development of HAV IgM (+) viral hepatitis A. From the second to the 14th day after admission to the hospital for mild jaundice of the skin and sclera in a 12-yr-old boy, the following neurological disorders have developed: absent Achilles and knee-jerk reflexes, diminished brachioradialis reflex, moderately decreased muscle power in the upper extremities and more pronounced power loss in the lower extremities. Facial palsy developed bilaterally, more expressed to the right. There was albuminocytologic dissociation of the cerebrospinal fluid and electrodiagnostic study showed findings compatible with the GBS subdivision - Acute inflammatory demyelinating polyneuropathy (AIDP). HAV could trigger GBS in the very beginning of liver inflammation in children. This insight may help wide range of medical professionals to early recognize and treat the peripheral neuropathy.

## Introduction

 Guillain-Barré (GBS) syndrome belongs to the group of peripheral immune-mediated neuropathies. Typical of the syndrome is the abruptly evolving polyradiculoneuropathy, as a rule, preceded by an inflammatory episode and likely attributable to an infectious agent. Extensive clinical and epidemiological evidence have been accumulated in support of the infectious origin of the condition: 75% of the patients with GBS reported a preceding infectious episode and in 30%-50% of them, there was serological evidence of infection (1). 

 GBS is rarely associated with hepatitis A virus (HAV) infection. Moreover, reports of GBS due to viral hepatitis A (VHA) in childhood are isolated. In the available literature, only a few cases of GBS with an antecedent episode of hepatitis A in children have been reported (2, 3). Here, we report an unusual case of GBS which occurrence coincided with the onset of VHA in a child. 

## Case report:

 A 12 yr old boy complained of general malaise, drowsiness, appetite loss, nausea, abdominal discomfort and darkening of the urine of two days duration. He was admitted to the Clinic of Infectious Diseases, St. George University Hospital, Bulgaria with mildly jaundiced skin and conjunctivas. 

Upon admission, the child was ambulatory and neurological signs and symptoms were undetected. On the following day jaundice intensified, malaise progressed, accompanied by abdominal pain, headache, and pain in the muscles of lower extremities. The child was afebrile and conscious. During the period from 2nd to 14th d after admission, the neurological examination revealed: absent Achilles and knee-jerk reflexes, diminished brachioradialis reflex, absent abdominal and cremasteric reflexes, moderately decreased muscle power in the upper extremities and more pronounced power loss in the lower extremities. The patient showed decreased mobility when lying in bed, and was unable to sit, stand and mobilize independently. Deep sensation was preserved, but superficial sensation was affected with paresthesia in the palms and soles. Facial palsy (House-Brackmann grade V) developed initially more expressed to the right, and Bell’s sign was positive bilaterally. Mouth movement was insufficient, but difficulty swallowing was absent as well as urine and fecal incontinence. Heart and respiratory rates, and peripheral arterial pressure were normal. 

There was albuminocytologic dissociation of the cerebrospinal fluid (CSF): normal pressure, normal cell count (2х10^6^/l), the sugar levels - 3.4 mmol/l (reference range 2.2-4.2 mmol/l), and about three times increased protein levels - 1.52 g/l (reference range 0.4-0.5 g/l). Treatment was initiated with intravenous immunoglobulin (IVIG) 0.400 gm/kg/ for 5 days. Over the following days, a favorable trend in the patient’s condition was observed with improved general medical condition and reduced pain syndrome. Regarding the peripheral neurological abnormalities, they reached a peak on day 14th post admission and after the brief plateau phase for about a week rapidly improved, so that on day 23rd the child was able to move independently, rise upright from a squatting position, and only absent deep tendon reflexes persisted. 

**Table 1:** Laboratory indicators of liver function and timeline of the neurological changes

**Day after admission ** **to hospital**	**Bilirubin levels** **(** **direct** **+** **indirect)** **Norm 21 μ** **с** **ml/l** c	**ALT** a **Norm 34U/L**	** AST** b **Norm 32U/L**	** Neurological signs and ** **Symptoms**
1st	36 (19+17)	1960	-	Undetected neurological abnormalities HAV IgM /+/ positive
2nd	-	-	-	Onset of neurological symptoms: pain in the muscles of lower extremities
3rd	95 (60+35)	2440	1816	Start with neurological signs: hypo and areflexia, power loss in the lower extremities, bilateral facial palsy;
5th	**-**	**-**	**-**	CSF: albuminocytologic dissociation Start of IVIG treatment
10th	90 (81+9)	1100	817	Full development of neurological signs and symptoms; electrodiagnostic changes;
14th	-	-	-	Stop of the neurological disorders; A plateau phase for a week;
23rd	19	55	29	The child is able to move independently
30th	17	33	**-**	Discharge from hospital with significant reversal of the peripheral neuropathy
4th month after discharge				Normal *tendon reflexes,* normal CMAP of the facial and peroneal nerves

^a^Alaninaminotransferase;

^b^Aspartataminotransferase;

^c^micromol per litre

On day 30th post admission the child was discharged clinically healthy in terms of the HAV infection, with significant reversal of the peripheral neuropathy. On the follow-up visits, he had no neurological deficit or electrodiagnostic changes on the fourth month after discharge.

Informed consent was obtained from the parents of the patient.

 The dynamics in the ALT, AST activity, and bilirubin level, as well as the timeline of the neurological changes, are displayed in Table 1. The remaining laboratory parameters were as follows: peripheral blood count, cholestatic enzymes, serum amylase, creatine kinase, choline esterase and electrolyte levels showed no abnormalities. Uro- and coproporphyrin tests were negative. Tests for Campylobacter jejuni, Epstein-Barr virus and Cytomegalovirus were negative. Nose and throat swabs were negative, as well as fecal cultures. The study of viral hepatitis markers /ELISA/ showed: HBsAg (-) negative, Anti HAV IgM (+) positive, HCV AB (-) negative. The child had received neither HAV vaccine nor prophylactic injection of immunoglobulin. He was contacted with other children with HAV during the large epidemics ongoing at that time in the Roma (Gypsy) headquarters in Plovdiv-city (Bulgaria).

 The electrodiagnostic testing - nerve conduction studies and needle electromyography (EMG) was performed on day 10 post admission and 5 day following CSF-examination, in the absence of fever at the beginning and throughout the child's disease process. The testing was conducted and the neurological disorders were further clarified in the University Pediatric Neurological Center. Here is a brief description of the electrodiagnostic findings derived from the patient's medical record: "Stimulation of the peroneus nerve bilaterally displayed a low amplitude of compound motor action potential (СМАP), prolonged distal motor latency, temporal dispersion and reduced nerve conduction velocity. Findings at facial nerve stimulation bilaterally showed: CMAP-mildly prolonged distal latency time, temporal dispersion and reduced rate of conduction. Sensory nerve conduction velocities and sensory action potentials were normal. The follow-up at 4 months post-admission documented normal *deep tendon reflexes* and normal motor activity of the child. The follow-up electrodiagnostic testing of the facial and peroneal nerves registered normal CMAP. Conclusion: The electrodiagnostic findings suggest acute inflammatory demyelinating polyneuropathy (AIDP)”.

## Discussion

Year 2016 marked the 100th anniversary of the original description of a benign polyneuritis with albuminocytologic dissociation in the cerebrospinal fluid. In 1990**, **the current diagnostic criteria for classic GBS were assessed (4). Later these criteria were reaffirmed, the electrodiagnostic standards were expanded and specific details added (5, 6)**, **therefore, the scale could be summarized as follows: 

**Features** **required** **for** **diagnosis**: “Progressive weakness of variable degree from mild paresis to complete paralysis in the arm and leg muscles; generalized hyporeflexia or areflexia” (6); 

**Features supportive of diagnosis**: Rapidly progression of symptoms from days to 90% motor weakness by 4 wk and stopping; relative symmetry of the neurological manifestations; mild sensory disturbances; involvement of cranial nerves, especially bilateral facial palsy; start of the healing process within 2-4 wk after stopping progression; autonomous dysfunctions - tachycardia, dysphagia, sweating and other vasomotor symptoms; lack of elevated temperature in the beginning and throughout the process; typical albuminocytologic dissociation of the CSF; typical electrodiagnostic features: lowered conductivity of peripheral motor neuron, more pronounced in the distal versus proximal segmen “Electrophysiology plays a determinant role in Guillain-Barré syndrome subtype classification” (6). Under the GBS rubric are included: acute inflammatory demyelinating polyneuropathy (AIDP), acute motor axonal neuropathy (AMAN), acute motor-sensory axonal neuropathy (AMSAN) and Miller Fisher Syndrome (7, 8). 

 The child in our report meets all the required and supportive diagnostic criteria of GBS in terms of his clinical features, CSF findings, and electrodiagnostic examination. Criteria that either cast doubt or rule out the diagnosis were carefully tested and consecutively excluded. “The acute inflammatory demyelinating polyneuropathy (AIDP), described in our report is the most frequent and easily recognizable form of GBS, accounting for nearly 90% of GBS-cases in most developed countries” (9). 

 GBS is characterized by inflammatory demyelination with or without secondary axonal involvement. In general, it is an acquired, predominantly motor immune-mediated polyradiculoneuropathy with activated T-lymphocytes and antibody response directed against the Schwann cells and myelin epitopes in the spinal paths and peripheral nerves. Pathologically, it correlates with multifocal reduction in the nerve conduction velocity to complete block, corresponding to the degree of axonal degeneration and nerve damage. The terminal nerves are most frequently affected - a fact that indicates involvement of antigen-antibody mechanisms in the pathogenesis of the disease (1, 7, 9).

Most studies showed an antecedent history of infection in patients with GBS (1-3). In our previous work, we have studied the literature (MEDLINE) and accounted no more than 16 patients of different ages who developed GBS after serologically proven VHA (HAV IgM /+/) (10). Only 3 of the cases were children, all of them were male. In 8 (50%) of these 16 cases, GBS clinically occurred one week after the onset of jaundice; at 4(25%) - after more than 7 d; аt 3(18.75%) - after 14 d and at 1(6.25%) after 21 d. The case we reported is unique in that the manifestations of peripheral neuropathy started too early, almost simultaneously with the clinical manifestations of hepatitis and developed along with the increase of jaundice. Considering, however, that the incubation period of VHA may be as long as 28 days (range: 15–50 d), still, our case may be called post-infectious with respect to hepatitis A virus, but ongoing regarding the liver inflammation onset and development. One could hypothesize the existence of common pathogenic mechanisms shared by both diseases. Nevertheless, authors emphasize the most often favorable prognosis of GBS in childhood (11). 

**In conclusion,** the association of GBS in children with VHA A is rare. The HAV can trigger GBS in the very beginning of the hepatitis signs and symptoms, the neurological disturbances progressing simultaneously with the progression of the liver inflammation. This can help pediatricians, infectious disease specialists, and wide range of medical professionals to early diagnose and treat the peripheral neuropathy.

## Author’s Contributions:

I. Baltadzhiev and N. Popivanova: Study Design

I. Baltadzhiev and I. Geneva: Data Collection

I. Baltadzhiev, I. Geneva and N. Popivanova : Data Interpretation

I. Baltadzhiev, I. Geneva and N.Popivanova: Manuscript Preparation

I. Baltadzhiev, I. Geneva and N. Popivanova: Literature Search

All authors read and approved the final manuscript.
